# Positive emotion inducement modulates cardiovascular responses caused by mental work

**DOI:** 10.1186/s40101-016-0116-4

**Published:** 2016-11-16

**Authors:** Xinxin Liu, Kazuma Ishimatsu, Midori Sotoyama, Kazuyuki Iwakiri

**Affiliations:** 1 National Institute of Occupational Safety and Health, 6-21-1 Nagao, Tama-ku, Kawasaki, Kanagawa 214-8585 Japan; 2 Graduate School of Health Care Sciences, Jikei Institute, 1-2-8 Miyahara, Yodogawa-ku, Osaka, 532-0003 Japan

**Keywords:** Positive emotion, Mental work, Blood pressure, Total peripheral resistance

## Abstract

**Background:**

Positive emotion is considered as an important factor related to health-relevant biological processes, including cardiovascular responses. To explore the possibility of using positive emotion as a tool to manage cardiovascular response of white-collar workers, we examined the influence on cardiovascular response of positive emotion inducement before a mental work.

**Method:**

Seventeen healthy males participated and performed a 10-min, PC-based Stroop color word task as their mental work. Before the task, 60 pleasant pictures chosen from the International Affective Picture System were presented in a random order under a positive emotion inducement condition while a gray screen was presented under a control condition. A 30-min relaxation period after completing the task was provided to examine the aftereffects of positive emotion inducement. Throughout these periods, systolic and diastolic blood pressure, mean arterial blood pressure, heart rate, stroke volume, cardiac output, and total peripheral resistance were measured continuously.

**Results:**

Blood pressure and total peripheral resistance were lower during the picture presentation period under the positive emotion inducement period compared to the control condition. All indices did not differ during the color word task period. During the relaxation period after the task, however, blood pressure and total peripheral resistance were lower under the positive emotion inducement condition compared to the control condition.

**Conclusion:**

Positive emotion inducement before a mental work beneficially modulates cardiovascular responses, suggesting that positive emotion inducement may be a useful tool to manage the cardiovascular response to mental work.

## Background

White-collar workers at risk for cardiovascular disorders (e.g., hypertension and ischemic heart disease) can be identified from their chronic exaggerated cardiovascular responses to mental work and delayed recovery after the work [1–4]. Some previous studies have reported that white-collar workers who have been exposed to cumulative work-related mental stress had a significant increase in systolic blood pressure (SBP), which is considered to be a long-term predictor of incident hypertension [2, 3]. Inhibition of exaggerated responses to mental work and promotion recovery of these responses are significantly important to manage work-related cardiovascular responses.

Cardiovascular responses include not only blood pressure but also the underlying hemodynamic aspects in increasing blood pressure. The hemodynamics are that mean arterial pressure (MAP) is elevated by cardiac output (CO) and/or total peripheral resistance (TPR) (MAP = CO × TPR), and CO is changed by heart rate (HR) and/or stroke volume (SV) (CO = HR × SV). The cardiac and vascular responses in increasing blood pressure are also considered to be risk factors of cardiovascular disorders, and exaggerated response in TPR was associated with high risk of hypertension [4–6]. Therefore, not only the magnitude of increasing blood pressure but also the underlying hemodynamics in increasing blood pressure should also be considered when examining prevention countermeasures of cardiovascular disorders.

As important factors for increasing hemodynamic responses (especially increases in MAP and TPR), negative emotions (such as anger, anxiety, and depression) are well documented [7, 8]. In recent years, some researchers began to pay attention to positive emotion as an important factor related to health-relevant biological processes, such as reduced hypothalamic-pituitary-adrenal (HPA) reactivity and promotion of a faster recovery of cardiovascular responses [9–14]. For example, Fredrickson and colleagues reported that low-arousal positive emotion (contentment and amusement) promoted heart rate return to baseline after a negative emotion-elicited task, and this effect was called the “undoing effect” [12, 13]. However, it is still unclear how positive emotion influences hemodynamic responses to mental work.

The previous studies about positive emotion mainly focused on the functions of positive emotions without a detailed classification of them, because positive emotions are not associated with specific actions and it is difficult to clearly classify a certain positive emotion from others [11, 12, 15–17]. Previous studies have reported some methods to induce positive emotions, such as presenting pleasant pictures or videos, listening to music and recalling happy experiences [12, 13, 15, 18]. In the present study, we presented low-arousal pleasant pictures to induce positive emotion before a mental work in order to investigate the influences of positive emotion inducement on cardiovascular responses. We examined cardiovascular responses using hemodynamic indices and hypothesized that positive emotion inducement before a mental work would beneficially modulate hemodynamic responses to a mental work.

## Methods

### Participants

Seventeen healthy males participated in this study. The ages, weights, heights, and BMIs of the participants (mean ± standard deviation (SD)) were 23.1 ± 1.7 years, 62.5 ± 10.0 kg, 172.4 ± 5.0 cm, and 21.0 ± 3.1, respectively. The participants were requested to refrain from exercise and alcohol intake on the night prior to the experiment and were prohibited from drinking caffeinated beverages or smoking during the 2-h period immediately preceding the experiment. After receiving a detailed description of the study, all participants gave written informed consent before taking part in this study. This study was approved by the Research Ethics Committee of the National Institute of Occupational Safety and Health in Japan (No. H24023).

### Mental work

The participants performed a 10-min, PC-based Stroop color word (CW) task as their mental work. The CW task involved a target word, the name of a color (e.g., green), which was printed in a different color from the meaning of the word (e.g., yellow). Around the target word (green), six buttons marked with the name of colors were presented (green, yellow, black, red, blue, and purple). The participants were instructed to press the button corresponding to the name of the target word’s color within 3 s (in the case of the example, the correct reaction would be to press the button marked “yellow”). If the participants pressed a wrong button or took over 3 s to press a button, the trial was recorded as an error, and then a new trial started automatically. The total number of trials was also recorded to examine task performance.

### Positive emotion inducement

Sixty high-pleasure, low-arousal pictures chosen from the International Affective Picture System (IAPS) were presented to induce positive emotion [19]. The IAPS provides pictures standardized on the basis of ratings of valence and arousal and the details of pictures used in the present study are shown in Table 1. The valence and arousal of each picture were measured by the Self-Assessment Manikin (SAM), which range from a smiling, happy figure to a frowning, unhappy figure when representing the valence dimension, and an excited, wide-eyed figure to a relaxed, sleepy figure when representing the arousal dimension [20]. The range of scores is from 1 to 9, with 1 being the most unpleasant or relaxed and 9 being the most pleasant or excited. For both valence and arousal, 5 is considered neutral. The valence of the chosen pictures ranged from 6.40 to 8.05, and the arousal ranged from 2.44 to 5.35, showing that these pictures were high pleasure with low-arousal. The categories of these pictures included 10 pictures of happy parents with children, 10 pictures of smiling infants, and 10 pictures of cute animals. The remaining 30 pictures were beautiful natural scenery (such as beaches, mountains, and flowers). Each picture was also evaluated by participants of the present study using the SAM (Table 1). The mean valence and arousal of all pictures were 5.84 ± 0.93 and 4.17 ± 0.65, respectively, and showed that the participants of the present study also evaluated these pictures as pleasure with low-arousal.

**Table 1:** The IAPS number of each picture and their valence and arousal scores

IAPS no.	IAPS (all)		IAPS (male)		Present study (male)		IAPS no.	IAPS (all)		IAPS (male)		Present study (male)	
	Valence	Arousal	Valence	Arousal	Valence	Arousal		Valence	Arousal	Valence	Arousal	Valence	Arousal
1410	7.00	4.17	6.86	4.00	7.05	4.25	2540	7.63	3.97	7.23	4.23	6.50	3.60
1440	8.19	4.61	7.96	4.76	6.45	4.60	2660	7.75	4.44	7.28	4.09	4.70	4.05
1441	7.97	3.94	7.71	3.84	7.80	2.90	5000	7.08	2.67	6.58	2.44	5.20	3.75
1463	7.45	4.79	7.10	4.46	7.25	5.55	5001	7.16	3.79	6.40	3.64	5.90	4.20
1604	7.11	3.30	6.40	3.17	5.60	4.20	5010	7.14	3.00	6.75	2.78	5.00	3.10
1610	7.69	3.98	7.28	2.82	6.65	4.25	5200	7.36	3.20	6.96	3.46	5.60	3.70
1721	7.30	4.53	6.85	4.58	6.40	4.75	5201	7.06	3.83	6.41	3.90	5.70	3.65
1722	7.04	5.22	6.85	4.65	6.85	5.15	5202	7.25	3.73	6.44	3.50	5.65	4.75
1731	7.07	4.56	6.84	4.64	7.35	4.25	5210	8.03	4.60	7.64	4.24	7.60	3.60
1740	6.91	4.27	6.81	4.81	5.05	5.80	5220	7.01	3.91	6.94	4.58	5.00	4.74
1750	8.28	4.10	7.89	4.21	6.45	4.55	5551	7.31	3.26	6.79	3.28	6.05	3.32
1920	7.90	4.27	7.83	4.21	6.65	4.70	5594	7.39	4.15	7.20	4.28	6.15	4.60
2040	8.17	4.65	7.63	4.33	4.85	3.90	5611	7.05	3.99	6.74	4.47	5.70	4.40
2045	7.87	5.47	7.43	4.66	4.15	4.47	5631	7.29	3.86	7.08	4.31	6.10	3.45
2050	8.20	4.57	7.80	4.05	4.15	4.26	5660	7.27	5.07	7.16	5.25	6.30	5.55
2057	7.81	4.54	7.16	4.32	4.75	4.10	5725	7.09	3.55	7.21	3.90	6.70	3.00
2058	7.91	5.09	7.34	4.47	4.25	3.95	5760	8.05	3.22	7.69	2.77	6.50	4.15
2070	8.17	4.51	7.69	4.02	4.75	4.75	5779	7.33	3.57	6.69	3.62	5.85	3.90
2071	7.86	5.00	7.45	4.60	5.15	3.95	5780	7.52	3.75	7.35	4.13	6.75	3.00
2080	8.09	4.70	7.56	4.35	4.75	3.90	5781	7.13	3.82	6.90	4.02	6.05	3.50
2091	7.68	4.51	6.99	4.20	6.65	3.95	5811	7.23	3.30	6.52	3.49	5.85	3.35
2151	7.32	4.37	6.52	4.17	5.55	4.05	5814	7.15	4.82	7.36	4.60	5.00	4.80
2153	6.98	4.40	6.63	4.55	5.15	4.65	5820	7.33	4.61	6.95	4.90	5.35	4.70
2160	7.58	5.16	6.87	5.31	4.90	4.20	5825	8.03	5.46	8.05	5.27	7.35	4.10
2165	7.63	4.55	6.74	3.89	3.85	4.65	5829	7.65	4.68	7.38	4.52	6.95	3.20
2260	8.06	4.26	7.63	3.74	5.00	3.95	5830	8.00	4.92	7.37	4.98	5.65	3.75
2310	7.06	4.16	6.61	3.89	4.95	4.30	5831	7.61	4.43	7.07	3.93	6.30	4.25
2311	7.54	4.42	7.24	4.85	5.35	4.90	5891	7.22	3.29	6.83	3.46	6.35	3.10
2332	7.64	4.30	7.18	4.20	5.85	4.10	7492	7.41	4.91	7.27	5.23	7.05	3.85
2340	8.03	4.90	7.65	5.35	6.20	5.10	7580	7.51	4.59	7.40	5.08	5.90	4.79

*IAPS* the International Affective Picture System, *all* all participants, including males and females, *male* only male participants, *valence* ranges from 1 (most unpleasant) to 9 (most pleasant), *arousal* ranges from 1 (most relaxed) to 9 (most excited)

### Protocol and parameters

**Fig. 1:**
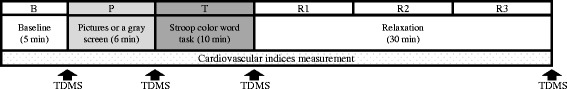
The protocol and measurement/analyzed periods. *TDMS* Two-dimensional Mood Scale, *B* the baseline, *P* the pictures or a gray screen presentation period, *T* the color word task period, *R1* the first 10-min relaxation period, *R2* the second 10-min relaxation period, *R3* the third 10-min relaxation period

The protocol is shown in Fig. 1. The participants rested quietly for at least 30 min after entering the laboratory, and then the recording sessions began. The measurement protocol consisted of a 5-min rest as a baseline (B), a 6-min pleasant picture or gray screen presentation (P), a 10-min CW task (T), and a 30-min relaxation period (R). Under the positive emotion inducement condition (EC), 60 pictures (each for 6 s) were presented in a random order, while a gray screen was presented under the control condition (CC). All participants took part in both conditions on different days and the order of the conditions was counterbalanced among the participants. Emotion state was evaluated by a Two-dimensional Mood Scale (TDMS) at the end of each recording period [21]. Systolic and diastolic blood pressure (SBP and DBP), mean arterial pressure (MAP), cardiac output (CO), heart rate (HR), stroke volume (SV), and total peripheral resistance (TPR) were measured continuously throughout all periods by a noninvasive continuous hemodynamic measurement monitor (Portapres Model-2, Finapres Medical Systems B.V.). This monitor measures beat-to-beat arterial pressure by a cuff worn on the fingers and computes SV from the arterial pressure according model flow method, then CO was calculated by SV and HR, and TPR was predicted from the mean pressure and model mean flow [22].

### Data analyses

The mean values of each period were calculated, while the final relaxation period was divided into three 10-min periods (R1, R2, and R3) in order to investigate the aftereffect as a function of the same time window. To avoid the influence of differences in the baselines, a change rate was calculated for each cardiovascular index by dividing a value in a period by its baseline (B). Two-way repeated measures ANOVAs [conditions × measurement periods] were conducted for all cardiovascular indices and emotion states. If Mauchly’s sphericity test was significant, Greenhouse-Geisser correction was used to estimate epsilon in order to correct the degree of freedom of the *F* value. Measures of effect size (partial *η* ^*2*^) and power were also reported. Multiple comparisons with the Bonferroni method were conducted to further examine the significant results. Paired *t* tests were conducted to compare task performances between conditions. The level of significance was set at 0.05 and significant tendency was set at 0.1. Statistical analysis was carried out using IBM SPSS Statistics 19 (IBM Corp.).

## Results

### Task performance

The total number of trials (mean ± SD) under the positive emotion inducement condition and under the control condition was 494.83 ± 77.31 and 492.61 ± 87.20, respectively. Error rates (error trials/total trials, by percent) (mean ± SD) under the positive emotion inducement condition and under the control condition were 2.01 ± 3.92, and 2.90 ± 4.76, respectively. There were no significant differences between conditions.

### Evaluation of emotion states

**Fig. 2:**
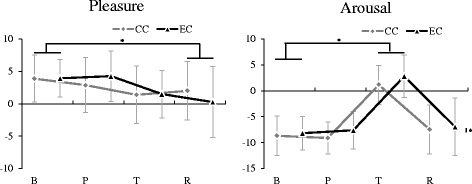
Subjective pleasure and arousal evaluated by Two-dimensional Mood Scale. Values are presented as the mean and standard deviation (SD). *CC* the control condition, *EC* the positive emotion inducement condition, *B* the baseline, *P* the pleasant pictures or a gray screen presentation, *T* the color word task, *R* the relaxation period; ^*^ *p* < 0.05

Emotional states evaluated by TDMS are shown in Fig. 2. The main effect of the measurement period was significant for pleasure (F(2.06, 32.97) = 6.15, *p* < 0.01, *η*_*p*_^*2*^ = 0.28, power = 0.87) and arousal (F(1.62, 25.97) = 46.65, *p* < 0.01, *η*_*p*_^*2*^ = 0.75, power = 1.00). The main effect of condition was significant for arousal (F(1, 16) = 5.41, *p* < 0.05, *η*_*p*_^*2*^ = 0.25, power = 0.59) but not significant for pleasure. Interaction between factors was not significant for both pleasure and arousal. The multiple comparisons showed that compared to the baseline, pleasure decreased significantly after relaxation (R < B, *p* < 0.05) and arousal increased significantly during the task period (T > B, *p* < 0.05). Arousal under positive emotion condition was significantly higher than under the control condition (CC < EC, *p* < 0.05).

### Cardiovascular responses

For MAP, the main effects of the measurement period (F(2.23, 35.75) = 27.67, *p* < 0.001, *η*_*p*_^*2*^ = 0.63, power = 1.00) and condition (F(1, 16) = 6.27, *p* < 0.05, *η*_*p*_^*2*^ = 0.28, power = 0.65) were significant. Interaction between factors was also significant (F(5, 80) = 4.04, *p* < 0.01, *η*_*p*_^*2*^ = 0.20, power = 0.94). SBP and DBP showed the same tendency as MAP and interaction between factors were also significant (SBP: F(5, 80) = 4.33, *p* < 0.01, *η*_*p*_^*2*^ = 0.21, power = 0.95; DBP: F(5, 80) = 3.55, *p* < 0.01, *η*_*p*_^*2*^ = 0.18, power = 0.90).

**Fig. 3:**
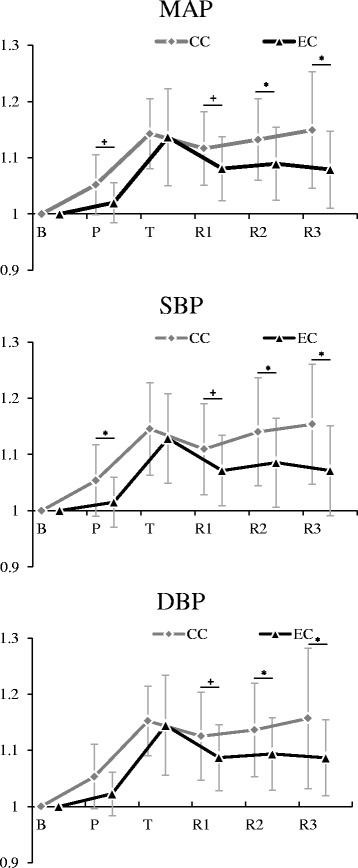
Comparison of blood pressure between conditions. Values are presented as the mean and standard deviation (SD). *CC* the control condition, *EC* the positive emotion inducement condition, *B* the baseline, *P* the pleasant pictures or a gray screen presentation, *T* the color word task, *R1* the first 10-min relaxation period, *R2* the second 10-min relaxation period, *R3* the third 10-min relaxation period, *SBP* change rate of systolic blood pressure from baseline, *DBP* change rate of diastolic blood pressure from baseline, *MAP* change rate of mean arterial pressure from baseline; ^*^ *p* < 0.05; ^+^ *p* < 0.10

Further analyses showed that MAP during picture presentation period was lower than during the gray screen presentation period (EC < CC, *p* < 0.10). During the task period, however, MAP did not significantly differ between conditions. In contrast, MAP were significantly lower under positive emotion inducement condition than under control condition during the relaxation period after the task (R1: EC < CC, *p* < 0.10; R2 and R3: EC < CC, *p* < 0.05) (Fig. 3). Under the control condition, MAP increased during the task period and did not change significantly during the relaxation periods after the task (T, R1, R2, and R3 > B, *p* < 0.05). Under positive emotion inducement condition, however, MAP increased during the task period but decreased immediately after the task and remained the same level during the last relaxation periods although it did not return to the baseline (T > R1, *p* < 0.05; T, R1, R2, and R3 > B, *p* < 0.05). SBP and DBP showed similar results as MAP (Fig. 3).

**Fig. 4:**
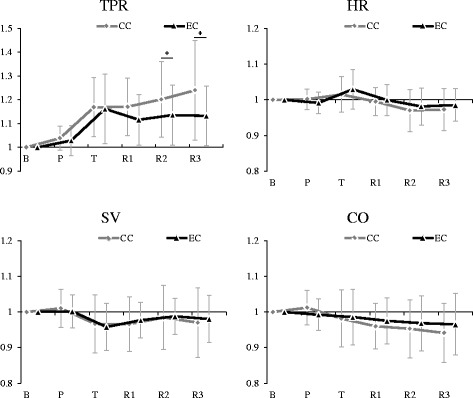
Comparison of cardiac and vascular responses between conditions. Values are presented as the mean and standard deviation (SD). *CC* the control condition, *EC* the positive emotion inducement condition, *B* the baseline, *P* the pleasant pictures or a gray screen presentation, *T* the color word task, *R1* the first 10-min relaxation period, *R2* the second 10-min relaxation period, *R3* the third 10-min relaxation period, *CO* cardiac output, *HR* heart rate, *SV* stroke volume, *TPR* change rate of total peripheral resistance from baseline; ^*^ *p* < 0.05; ^+^ *p* < 0.10

The underlying hemodynamic responses in increasing MAP are shown in Fig. 4. TPR showed a similar change tendency as MAP. The main effect of the measurement period (F(2.29, 36.58) = 19.06, *p* < 0.001, *η*_*p*_^*2*^ = 0.54, power = 1.00) and interaction between factors (F(2.51, 40.22) = 3.25, *p* < 0.05, *η*_*p*_^*2*^ = 0.17, power = 0.65) were significant. Further analyses showed that TPR during relaxation periods were lower under the positive emotion inducement condition than under the control condition (R2 and R3: EC < CC, *p* < 0.10) (Fig. 4).

The cardiac responses did not significantly differ between conditions, and the interaction between factors was not significant (Fig. 4). The main effect of the measurement period was significant for HR (F(2.07, 33.19) = 5.31, *p* < 0.01, *η*_*p*_^*2*^ = 0.25, power = 0.81), SV (F(2.46, 39.28) = 3.56, *p* < 0.05, *η*_*p*_^*2*^ = 0.18, power = 0.69), and CO (F(2.79, 44.58) = 4.82, *p* < 0.01, *η*_*p*_^*2*^ = 0.23, power = 0.86). Multiple comparisons showed that compared to the baseline, HR increased during the task period (T > B, *p* < 0.10) but decreased during the relaxation periods (R2 and R3 < B, *p* < 0.05); SV decreased during task period and the first relaxation period (T and R1 < B, *p* < 0.05) and CO decreased during relaxation periods (R1, R2, and R3 < B, *p* < 0.05).

## Discussion

Previous studies suggested that positive emotion inducement promoted heart rate returning to baseline after a negative emotion-elicited task [12, 13]. Based on these, we hypothesized that positive emotion inducement before a mental work would beneficially modulate hemodynamic responses. The results of the present study showed that MAP were lower during the picture presentation period and suggested that the blood pressure was modulated by positive emotion inducement before the mental task. During the task period, however, MAP increased significantly for both conditions and did not differ between conditions. This result was considered that the influences of positive emotion inducement may be masked by the task itself because the task performance was not different between conditions. In contrast, MAP during the relaxation period after the task was significantly lower under the positive emotion inducement condition compared to the control condition. Under the control condition, MAP did not change during the relaxation periods after the task, and this tendency agreed with our previous studies, which suggested that blood pressure did not show a decreased tendency immediately after mental tasks [23–25]. Under the positive emotion inducement condition, however, MAP decreased immediately after the task and TPR showed a similar response tendency, although they did not return to the baseline during the last measurement periods. These results suggested that positive emotion inducement before a mental task beneficially modulated the hemodynamic responses during the relaxation period after the task, resulting in a quicker recovery in hemodynamic responses compared to control condition without affecting task performance.

On the other hand, cardiac responses (HR, SV, and CO) did not differ between conditions. A previous study has reported that positive emotion inducement after a negative emotion-elicited task promoted heart rate return to baseline [12]. The present study presented pleasant low-arousal pictures to induce positive emotion before a mental task and we think that the different timing and characteristic of task may influence the effects on cardiac responses. Previous studies have reported that positive emotion inducement may influence automatic nervous and endocrine system responses [9, 11, 18, 26–28], but the physiological mechanism of influences of positive emotion inducement on hemodynamic response is not clear yet, and we think further examination is necessary in the future.

To induce positive emotion, we presented 60 pleasant with low-arousal pictures chosen from IAPS during a 6-min period before a mental task in the present study. The pictures were also evaluated as pleasant with low-arousal by the participants of the present study. Fredrickson et al. used films of waves breaking on a beach and a small dog playing with a flower, and they defined the elicited emotions as contentment and amusement [12]. We think that the pictures used in the present study also included contentment, amusement, and so on. In contrast, the emotion state evaluated by the Two-dimensional Mood Scale was not significantly different for pleasure between conditions. The Two-dimensional Mood Scale did not evaluate positive emotion directly but calculated pleasure and arousal scores from eight adjectives about vitality and stability [21]. The indirect evaluation structure may influence the sensitivity of detecting changes of positive emotion. On the other hand, the influence of positive emotion inducement on cardiovascular responses may not always depend on the subjective sensation of participants.

As an initial-stage study investigating the effects of positive emotion inducement on cardiovascular responses, the results of the present study demonstrated that a short-term positive emotion inducement (6 min) before a mental task beneficially modulated hemodynamic responses without affecting task performance. These results suggested the possibility of inducing positive emotion (i.e., looking at pleasant and relaxing pictures) during short breaks or rests during working hours may beneficially modulate cardiovascular responses. On the other hand, because the experiment design was different so far from the real work situation, there are also many works to do before using positive emotion inducement as a tool to manage cardiovascular response of white-color workers, including examination longer task periods.

There are also some limitations in the present study. First, because we used pleasure pictures with low-arousal to elicit positive emotion, the results of the present study may be limited and did not apply for positive emotions with high arousal. Second, only one task was used in the present study. Our previous study reported that different types of tasks have different hemodynamic response characteristics: a negative emotion-elicited task caused a higher vascular response, but the intellectual mental task caused both cardiac and vascular responses [29]. The effects of positive emotion inducement may vary among different types of tasks, and more tasks should be examined in the future. Finally, whether other positive inducement methods also have similar effects and whether there is interaction between the inducement methods and tasks, are unknown. All of these factors should be examined further in the future.

## Conclusions

The present study demonstrated that positive emotion inducement before mental work beneficially modulates hemodynamic responses without affecting task performance, suggesting the possibility of using positive emotion inducement as a tool to manage cardiovascular responses to mental work.

## Abbreviations

ANOVA: Analysis of variance
B: Baseline
CC: Control condition
CO: Cardiac output
CW: Stroop color word task
DBP: Diastolic blood pressure
EC: Positive emotion inducement condition
HR: Heart rate
IAPS: The International Affective Picture System
MAP: Mean arterial pressure
P: A 6-min picture or gray screen presentation
R: A 30-min relaxation period after the task
R1: The first 10-min relaxation period
R2: The second 10-min relaxation period
R3: The third 10-min relaxation period
SAM: The Self-Assessment Manikin
SBP: Systolic blood pressure
SD: Standard deviation
SV: Stroke volume
T: A 10-min color word task period
TDMS: Two-dimensional Mood Scale
TPR: Total peripheral resistance
